# CD47 as a Potential Target to Therapy for Infectious Diseases

**DOI:** 10.3390/antib9030044

**Published:** 2020-09-01

**Authors:** Lamin B. Cham, Tom Adomati, Fanghui Li, Murtaza Ali, Karl S. Lang

**Affiliations:** Institute of Immunology, Medical Faculty, University of Duisburg-Essen, Hufelandstr. 55, 45147 Essen, Germany; wennatom@yahoo.com (T.A.); 15927580042@163.com (F.L.); murtaza142426@st.jmi.ac.in (M.A.); KarlSebastian.Lang@uk-essen.de (K.S.L.)

**Keywords:** anti-CD47, macrophages, dendritic cells, T cells, therapy, viral infection

## Abstract

The integrin associated protein (CD47) is a widely and moderately expressed glycoprotein in all healthy cells. Cancer cells are known to induce increased CD47 expression. Similar to cancer cells, all immune cells can upregulate their CD47 surface expression during infection. The CD47-SIRPa interaction induces an inhibitory effect on macrophages and dendritic cells (dendritic cells) while CD47-thrombospondin-signaling inhibits T cells. Therefore, the disruption of the CD47 interaction can mediate several biologic functions. Upon the blockade and knockout of CD47 reveals an immunosuppressive effect of CD47 during LCMV, influenza virus, HIV-1, *mycobacterium tuberculosis*, *plasmodium* and other bacterial pneumonia infections. In our recent study we shows that the blockade of CD47 using the anti-CD47 antibody increases the activation and effector function of macrophages, dendritic cells and T cells during viral infection. By enhancing both innate and adaptive immunity, CD47 blocking antibody promotes antiviral effect. Due to its broad mode of action, the immune-stimulatory effect derived from this antibody could be applicable in nonresolving and (re)emerging infections. The anti-CD47 antibody is currently under clinical trial for the treatment of cancer and could also have amenable therapeutic potential against infectious diseases. This review highlights the immunotherapeutic targeted role of CD47 in the infectious disease realm.

## 1. Introduction

The CD47 is expressed on both hematopoietic and non-hematopoietic cells and plays crucial role in immune regulation and maintenance of homeostasis [1,2,3]. Its expression level varies depending on cell types and pathophysiological conditions. In normal physiological conditions, all healthy cells express moderate level of CD47. However, migrating stem cells and young red blood cells exhibit increased levels of CD47 to evade macrophage attack [4,5,6,7,8]. In pathologic conditions, varieties of cancer cells are known to induce increased CD47 surface expression as a mechanism to evade macrophage mediated phagocytosis [9,10,11]. In addition to cancer, all immune cells upregulate CD47 upon pathogen invasion [12]. Typically, poxvirus has been shown to encode expression of CD47-like protein and this strongly inhibits the activation of macrophages and T cells. Thus, the CD47 expression in poxvirus is an immune evasion mechanism to promote its virulence and pathogenesis [13,14].

The two most known interactions of CD47 is with signal regulatory protein alpha (SIRPa) and thrombospodin-1 (TSP-1). SIRPa is widely expressed on macrophages and dendritic cells while thrombonspondin-1 is a secreted matricellular glycoprotein [12]. The CD47-SIRPa interaction induces an antiphagocytic signal in macrophages and dendritic cells. This interaction leads to the recruitment and activation of Src homology phosphate (SHP-1 and SHP-2) thereby inhibiting the myosin-IIA and this results in prevention of phagocytosis [12,15]. The mechanism of CD47-mediated suppression on innate immune cells is via the recruitment and activation of Src homology two domain-containing phosphatases, SHP-1 and SHP-2. Activated SHP-1 and SHP-2 dephosphorylate immune-receptor tyrosine-based inhibitory motifs (ITIMs), thereby preventing downstream activation-signaling in macrophages, dendritic cells and NK cells [16,17,18,19,20,21,22,23]. The disruption of CD47/SIRPa-signaling increases macrophage mediated phagocytosis of diseased vascular tissue and cancer cells. This leading to regression of much different type of tumors both in vivo and in vitro [24,25,26,27,28,29]. Similar to cancers, there is growing evidence that the inhibition of CD47/SIRPa interaction induces an antimicrobial effect during infections [30,31,32,33].

Thrombospondin-1 is a multifunctional protein that plays several crucial roles in regulating cell proliferation and differentiation. Although it is shown to bind to multiple proteins, the most studied interaction is with CD47 [34,35]. The CD47-TSP-1 interaction on antigen presenting cells (APCs) inhibits inflammasomes and interleukin 1b (IL-1b) and also inhibits T cells proliferation, activation and cytotoxicity [36]. In addition to overexpression of CD47 by cancer cells as a survival mechanism, TSP-1 ligation of CD47 is shown to increase proliferation and survival of cutaneous T-cell lymphomas (CTCL) and hence blockade of CD47/TSP-1 interaction results in CTCL regression [37,38].

As an inhibitory innate immune checkpoint molecule, the CD47-signaling pathways have been found to be an important in regulating both innate and adaptive immunity. The blockade of CD47 using anti-CD47 antibody shows a promising therapeutic effect on different tumors. Indeed, several studies on the genetic inactivation or blockade of CD47 demonstrate therapeutic potential during infection. Recently, Cham et al. Shown that CD47 blockade mediates similar immunotherapeutic effect during viral infection due to increased activation of both innate and adaptive immune response after CD47 blockade. However, the use of anti-CD47 antibody in other infections remains to be investigated. In this review, we recapitulate anti-CD47 antibody as a potential therapeutic target for infectious diseases.

## 2. CD47 Expression and Infection

The level of CD47 expression determines the susceptibility of target cells to be licensed for destruction by macrophages. For instance, young red blood cells (RBCs) are protected from phagocytosis due to increased level of CD47 expression while the loss of such antiphagocytic protein on older RBCs leads to their rapid clearance [8,39,40,41,42,43,44,45,46,47]. It is not by coincidence that cancer cells are also associated with increased CD47 expression. Different cancers have different levels of CD47 expression, however, cancers with higher CD47 expression are more resistant to phagocytosis and anti-CD47 immunotherapy [48]. Mechanistically, TNFa-NFkB1-signaling pathway directly regulates CD47 expression suggesting that cancers can evolve to drive CD47 overexpression to escape immune surveillance [49]. Thus, CD47 overexpression on cancers is a crucial survival mechanism.

Similar to cancers, poxvirus has been recently shown to have evolved similar mechanism by encoding a CD47-like protein to evade host immune defense. The expression of decoy CD47 by poxvirus inhibits innate and adaptive immune cells and this contributes to its virulence. The depletion of CD47 in this virus results in loss of its pathogenicity [13]. In addition to poxvirus, herpesvirus and adenoviruses similarly encode CD47 expression potential and other immunoglobulin superfamily genes to undermine the host immune defense in order to guarantee their survival [14]. However, whether other viruses, bacteria, fungi and parasites (over)express CD47 remains unknown.

Recently, Tal et al and Cham et al reveals from their different modes of infections an upregulation of CD47 during in LCMV, Friend virus, VSV, HCV, HIV, La Crosse virus, SARS-CoV2, *mycobacterium tuberculosis* and other infections [12]. Unlike cancers, CD47 upregulation during infection stems from cytosolic or endosomal stimulation of pattern recognition receptors. The presence of certain inflammatory cytokines such as interferon alpha, tumor necrosis factor alpha and CxCCl-10 in the serum of chronically infected patients can induce CD47 upregulation in naïve immune cells in vitro. *Salmonella typhi* lacking potent inducer of TLR was found to have reduced CD47 expression during infection suggesting that TLR activation is important for CD47 upregulation during infection. The upregulation of CD47 as an immune-inhibitory molecule post the question on why the host dampen its defense during cancer and infection. However, the role of CD47 indicates that CD47 help to prevent over activation of immune cells that may lead to an immune-pathology [50]. CD47 is therefore important to balance the proinflammatory and anti-inflammatory signals. Indeed, we show that the virus infected cells induce increased CD47 level [12]. However, whether virus and other infectious agents can directly induce the infected cell to increase its CD47 expression to evade immune surveillance remains to be understood.

## 3. Role of CD47 in Immune Response

The role of CD47 as a regulatory molecule was initially identified in red blood cells; however, several studies have shown CD47 to play crucial roles in homeostasis, immune regulation, stress, tumor, infection, atherogenesis, etc. CD47 has high binding affinity to SIRPa, TSP-1 and other integrin molecules on platelets, sickle red blood cells, microglia, B lymphocytes, etc. [51,52,53,54,55]. Due to the varieties of binding molecules, the type of signal induce by CD47 depends on the type of ligand and the condition on which the binding occur. For instance, CD47-SIRPa can mediate an antiphagocytic signaling on phagocytic cells, CD47–TSP-1interaction can promote angiogenesis and modulate nitric oxide-signaling [56]. However, most studies unanimously show an antiphagocytic and immune-modulatory function of CD47.

### 3.1. CD47 and Innate Immunity

One of the primary role of CD47 is highlighted by its key function in immune cell migration. CD47 was initially shown to be crucial in the migration of neutrophils, dendritic cells and other leukocytes [57,58,59]. With such a role, one may speculate that the use of anti-CD47 could comprise host immunity. However, several recent studies on the use of anti-CD47 in both cancer and infections contradict this speculation. The most studied role of CD47 is the antiphagocytic function. The deficiency of CD47 is known to increase activation of macrophages to phagocytose the target cells. However, there are growing evidences that the lack of CD47 on dendritic cells can also increases their phagocytic rate, antigen processing and antigen presentation to adaptive immune cells [12]. The interaction of CD47 on T cells with TSP-1 or SIRPa on dendritic cells inhibits immune cell maturation and inflammatory response such as cytokine production [60]. Therefore, CD47-signaling on dendritic cells is crucial for effective immune response in inflammatory conditions such as cancers and infections. The blockade of CD47 has also shown comparable activation of both macrophages and dendritic cells. Similar to phagocytic cells, lack of CD47 increases NK cells activation and cytotoxicity. However, the administration of anti-CD47 does not show any effect on NK cells response [50]. Therefore, expression of CD47 on all innate immune cells plays a negative regulatory role.

### 3.2. CD47 and Adaptive Immunity

On T cells, the CD47 interaction with either SIRPa or TSP-1 may also suppress T cells proliferation and activation [61,62]. Disruption of CD47 interaction on T cells has been shown to increase T cells response in both tumor and viral infection [12,63]. However, the increased activation and priming by antigen presenting cells after CD47 blockade is likely to be the major reason for the observed T cells phenotype. Additionally, the disruption of CD47 interaction on T cells leads to increased potency of CD8 T cells as measured by interferon–gamma production. The treatment of anti-CD47 on naïve T cells showed an enhanced differentiation into regulatory T cells [24]. Therefore, the blockade of the CD47 interaction on T cells can lead to an increased effector and regulatory T cells response. Cham et al. recently show that the administration of CD47 enhances both T cells effector and regulatory functions during LCMV and HIV infection. Similar to T cells, the CD47 on B lymphocytes is also shown to interact with SHPS-1 and resulting inhibition of B cells response [64,65]. The genetic inactivation of CD47 on B cells results in enhanced antibodies B cells response during influenza virus infection [33]. To account for the importance of CD47 on both innate and adaptive immune response, one can suggest that CD47 plays an immune-suppressive role by regulating inflammatory responses in patho–physiological conditions.

## 4. CD47 Genetic Inactivation and Infection

**Figure 1:**
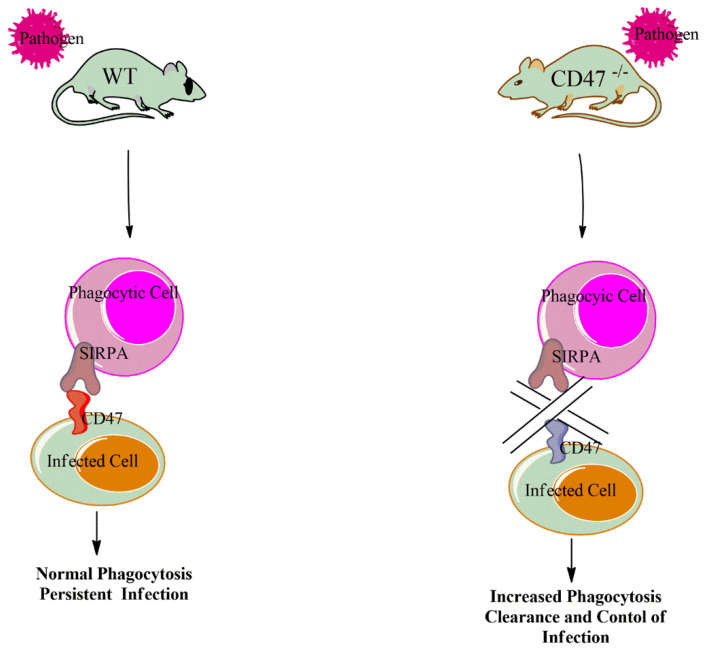
Interaction of CD47-SIRPa following an infection limits the phagocytosis of infected cells (e.g., *plasmodium-infected* red blood cells (RBCs)) and result to persistence of infection. On the contrary, the genetic inactivation of CD47 leads to an increased phagocytosis of (e.g., *plasmodium*) infected cells and faster control of infection.

Most studies on the role of CD47 during infection employed the used of CD47 knockout mice [30,32,33,66,67]. All these studies unanimously points out the immunosuppressive role of CD47 upon infection. However, CD47 plays both host protective and detrimental role depending on the type of infection. The deficient of CD47 during *plasmodium falciparum* infection results in increased phagocytosis, reduced parasitemia, reduced endothelial activation, increased percentage of splenic F4/80 macrophages and increased survival rate as demonstrated in Figure 1. The increased CD47 expression on infected red blood cells is protects the *plasmodium* from phagocytosis and compliment mediated destruction and clearance. The CD47 also modulates the nitric oxide synthase which is reported to be crucial during *plasmodium* infection. Therefore, suggesting an important role of CD47-SIRPa interaction in innate control of malaria. Together, these findings suggesting that the administration of CD47 neutralizing antibody can result in a significant reduction of malaria parasites and apparently leading to faster infection control [30,32]. In two distinct bacterial infections, the deficiency of CD47 protects mice from *E.coli* pneumonia and LPS-induced acute lung injury. Similar to plasmodium infection, the increased phagocytosis of the bacteria-infected cells by macrophages and neutrophils in the knockout mice led to protection during these infections as illustrated in Figure 1. The CD47 knockout mice displayed a reduced pulmonary lung edema, reduced bacteremia, increased neutrophil infiltration, therefore, suggesting that CD47 could be a potential target for the treatment of acute lung injury [31,66]. Beside bacterial and parasitic infections, lack of CD47 enhances virus specific neutralizing antibodies response during influenza virus infection. The CD47 deficient mice were better protected following influenza virus vaccine [33]. On the contrary, the lack of CD47 was shown to be detrimental during LCMV-Armstrong infection. This is as result of an increased activation and effector function of NK cells in the knockout mice. These hyper-activated NK cells in the knockout mice leads to increase killing of antiviral T cells thereby reducing the CD8 T cells numbers in the CD47 knockout mice during LCMV-chronic infection. Considering the crucial role of CD8 T cells in clearing and controlling LCMV infection, one can speculate that the poor LCMV control after CD47blockade is attributed to reduced T cell numbers in the CD47^-/-^ mice [50]. However, the genetic inactivation of some proteins (such as CD47) in certain regulating immune cells such as NK cells can exert varying effects. For example, unlike in the genetic inactivation, the blockade of CD47 shows an increased proliferation, activation and cytotoxicity of antiviral CD8 T cells and results in faster LCMV control [12]. As an immunosuppressive molecule, the lack of CD47 led to elevated neutrophils, macrophages and inflammatory cytokines level during candida albicans infection. This enhanced immune response surprisingly led to wide dissemination of the infection, resulting in increased morbidity and mortality in the CD47 knockout mice [67]. Therefore, CD47 is an immune checkpoint molecule that can regulate both innate and adaptive immunity thereby influencing the outcome of an infection. With such an effect during infection, the blockade of CD47 using anti-CD47 antibody could be a potential therapeutic target for wide range of infections. Tal et al. recently show an impressive CD47 deficiency associated antimicrobial effect during mycobacterium tuberculosis and other bacterial pneumonia infections. Therefore, there is a potential to rationale CD47 blockade in treating infections due to bacteria and multidrug resistant bacterial strains.

## 5. CD47-Blocking Antibodies

To date, several studies have been done using several anti-CD47 antibodies in various cancer studies with insightful mechanisms of action. CD47 expression become an interesting research area when cancer cells were known to have an increased CD47 expression as a survival mechanism and the blockade of CD47 resulted in increased phagocytosis by macrophages and suppression of many tumors.

Due to the increased surface expression of CD47 on cancer cells, anti-CD47 can selectively target a macrophages mediate phagocytosis and clearance. It is important to understand that blocking an antiphagocytic molecule such as CD47 may not be enough for phagocytosis. The expression of several pro-phagocytic ligands or molecules on the surface of stressed cells, tumor, and infected cells is important to facilitate phagocytosis. Pro-phagocytic molecules such as phosphatidylserine, asialoglycoproteins and migration of calreticulin from the endoplasmic reticulum to cytoplasmic membrane occur on tumor and infected cells unlike in healthy cells [68,69,70]. Considering the fact that healthy cells does not or express these pro-phagocytic molecules at low level, therefore the administration of anti-CD47 may not cause phagocytosis or apoptosis on healthy cells.

Several different types of anti-CD47 antibodies such as MIAP301, MIAP410, Hu5F9-G4, CC-90002, SRF231, B6H12.2, ALX148 has been use in during cancer and infection [12,15,25,71,72]. The two most known clones of anti-mouse CD47-blocking antibodies are MIAP410 and MIAP301. The MIAP410 treatment in virus infected mice is reported to result to an increased proliferation and activation of innate and adaptive immune cells. This antibody has been reported to have both antiviral and anti-tumor in two distinct viral infections and several tumors. The Hu5F9-G4 is a humanized anti-CD47 that disrupts the CD47-SIRPa interaction thereby promoting macrophage mediated phagocytosis and elimination of several human tumors both in vivo and in vitro. Due to the unselected inhibition of CD47, the administration of anti-CD47 antibody leads to a mild anemia as a result of increased red blood cells attack by phagocytic cells [71,73,74]. The CC-90,002 is also a humanized anti-CD47 Abs that is reported to be under clinical trial for the treatment of both solid and hematologic malignancies [75]. Another humanized antibody (SRF 231) is also reported to be tested on pre-clinical trial for malignancies [15]. The B6H12.2 CD47 blocking antibody has been use to suppress non-Hodgkin lymphoma tumor [72,76]. The ALX148 is a more modified version of CD47 blocking antibody by fusing the inactivated human IgG1 Fc with the modified SIRPa D1 domain. This antibody has been reported to promote macrophages and dendritic cells activation, increased inflammatory cytokine production and increased antitumor T cells effector function. Addition to its effective antitumor function, the antibody does not cause anemia in an in vivo setting [77]. All these CD47-blocking antibodies (both mouse and humanized) unanimously show a similar immunological effect resulting in increased phagocytosis and activation of antigen presenting cells, increased proliferation and activation of cytotoxic CD8 T cells during tumor and viral infections.

## 6. CD47 Blockade and APCs Activation During Infection

As an inhibitory protein, both the genetic knockout and blockade of CD47 results in increased activation of antigen presenting cells (APCs) during cancer and infection [74,78]. Several studies reveal that the blockade of CD47 as an antiphagocytic ligand increase the phagocytic activity and thereby enhancing antigen presentation to T cells [10,52,79,80]. Therefore, treatment with anti-CD47 antibody increases the capacity of the APCs in bridging innate and adaptive immune response leading to enhanced potency of T and B cells immunity. However, there are growing evidences that the blockade of CD47 ligation can induce cancer cell apoptosis [81]. In addition to above-mentioned effect, the blockade of CD47 enhances interferon-I response and this was strongly associated with upregulation of STING pathway in APCs in tumor [82,83,84]. Demeure et al. suggests that the disruption of ligation of CD47 during pathogen invasion may increase inflammatory cytokine response by dendritic cells [85]. Though not well studied, CD47 was identified as an interferon stimulated gene and as a host defense mechanism upon IFNa stimulation [86]. With such an immunotherapeutic effect of anti-CD47 antibody, one could speculate that the treatment of the anti-CD47 may also increase the interferon response and APCs activation during infection.

**Figure 2:**
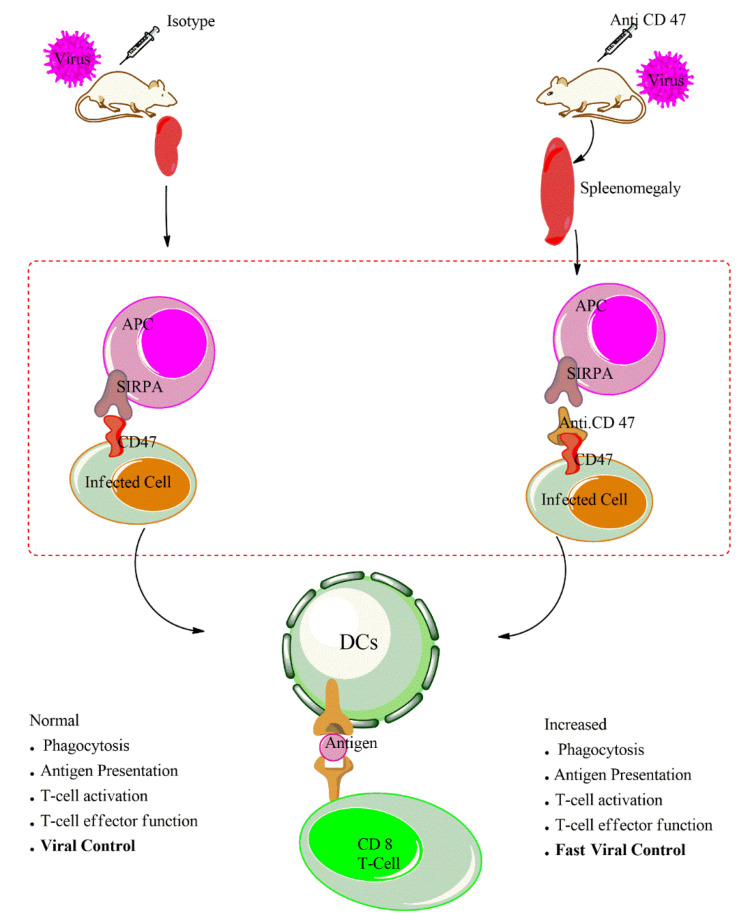
Treatment of isotype control to mice following a viral infection leads to limited change in the splenic architecture, normal phagocytosis, normal antigen presentation and therefore resulting to a normal activation and cytotoxicity of T cells. On the other hand, the administration of anti-CD47 antibody result to a splenomegaly, increases phagocytosis of infected cells, increase antigen presentation, enhances T cell activation and function and faster viral control.

Our recent study provides evidence that the administration of anti-CD47 leads to increased macrophages proliferation, infiltration and uptake of virus and/or virus infected cells by macrophages during LCMV infection. The treatment of anti-CD47 enhances the activation of antigen presenting cells as indicated by surface expression of activation markers such CD86 and CD80 on macrophages and dendritic cells. Cham shown that in vivo administration of anti-CD47 in wild type during LCMV infection increases et al. APCs activation as illustrated on Figure 2. To further determine whether macrophages and/or dendritic cells are responsible for the antibody mediated increased activation of adaptive immune response, we depleted the macrophages using clodronate. The depletion of CD11b^+^ macrophages followed by anti-CD47 treatment can still enhance T cells response. On the contrary, the depletion of dendritic cells using the CD11c-DTR mice followed by anti-CD47 treatment shows impaired T cells response and persistence of LCMV virus replication in serum and all organs. This indicates that macrophages may an essential innate immune cell mediating the anti-CD47 phenotype at an early time point. However, dendritic cells and not macrophages were the most crucial cells in bridging and activation of the robust CD8 T cells immune response. In vitro, dendritic cells—CD8Tcells co-culture with anti-CD47 treatment reveals that blockade of CD47 in vitro can enhance dendritic cells activation in priming of CD8 T cells. This increased APC activation status in absence of CD47-signaling amplifies T cell activation and cytotoxicity during viral infection [12]. It is tempting to speculate that with such an increased APC activation, this could enhance neutralizing antibody response in a similar manner.

## 7. CD47 Blockade and T Cell Function During Infection

Most cancer studies reveal that an increased antitumor cytotoxic CD8 T cells is crucial for tumor regression. Similar to cancers, the clearance and control of many important human acute and chronic infections depends on CD8 T cells response [87,88,89]. Therefore, an immunotherapy that results to an enhanced T cell immunity would be important against cancers and infections.

The anti-CD47 administration has shown to increase antitumor T cell-mediated elimination of many different tumors [24,36,38]. However, the findings from these studies suggest that that the anti-CD47 immunotherapy increases CD8 T cell cross-priming by dendritic cells not by macrophages. Additionally, the blockade of CD47 interaction suppresses tumor growth due to increased T cell function and thereby decreasing other suppressive immune markers in human head and neck squamous cell carcinoma [90,91].

The T cell immune response particularly cytotoxic CD8 T cells mediate essential antiviral effect during LCMV infection and other important human persistent infections such as hepatitis B virus (HBV), hepatitis C virus (HCV), human immunodeficiency virus (HIV), etc. [89]. It is well known and established that CD8 T cells are key in LCMV clearance and control. During LCMV infection, the treatment of anti-CD47 enhances the proliferation, activation and potency of antiviral T cells and led to faster clearance and control of acute and chronic LCMV infection. Similarly, the blockade of CD47 in humanized mice infected with HIV-1, reduced the p24 HIV antigen and restored both CD4 and CD8 T cells counts to a level comparable to health uninfected mice. The depletion of CD8 T cells lead to impaired virus control comparable in both antibody treated and isotype, indicating that the effect of the antibody mediated virus clearance was CD8 T cells dependent. Furthermore, the CD47 blockade were not acting directly through SIRPα on the T cells, suggesting that the effect of anti-CD47 antibody on CD8^+^ T cells was mediated via the better activation of antigen presenting cells [12]. The capacity of the antibody to mediate similar immunological response in two distinct virus models shows that the anti-CD47 blockade can have broad applicability to certain important human viral infections whose clearance and control is T cells mediated as demonstrated in Figure 2. With such an immunological boosting effect, other possible administration of CD47 blockade may include viruses such as, varicella zoster virus, human papilloma virus, cytomegalovirus, Epstein–Barr virus, Ebola virus, SARS-CoV-2, etc.

Similar to the effect of CD47 knockout in influenza virus, some of our unpublished findings indicate that the treatment of anti-CD47 in the VSV infection show an increased VSV neutralizing antibody response in the antibody treated mice. These mice also had a better survival rate compare to control mice after an infection of a lethal dose. This findings indicate that the anti-CD47 dose not only activate T cells, but also B cells response and will be vital to most viral infection whose clearance and control is mediated by B cells. This indicates the potency of the antibody in the enhancement of vaccination.

## 8. Conclusions

As highlighted above, both the genetic knockout and blockade of CD47 as an immunosuppressive protein leads to an elevated innate and adaptive immune responses. The administration of anti-CD47 antibody increases the phagocytic activities of virus infected cells, increases antigen processing and presentation by APCs and this enhances activation capacity of antiviral CD8 T cells. Therefore, the anti-CD47 antibody increases the activation of both innate and adaptive immune response in two distinct viral infections. With such an immune-therapeutic effect of anti-CD47 blockade on both cancer and viral infection, this antibody would be a unique target in virus-associated cancers such human papillomavirus, hepatitis B and hepatitis C virus, Epstein–Barr virus, human T-lymphotropic virus, Kaposi’s sarcoma-associated herpesvirus (KSHV) and (re)emerging infections. The anti-CD47 is currently under clinical trial for cancer and can be amenable as an immunotherapeutic target for infectious diseases.
